# West Ham Hospital, Stratford, E.

**Published:** 1893-11-04

**Authors:** 


### WEST HAM HOSPITAL, STRATFORD, E.

"Mr. Pagensteoher, the secretary, writes: This hospital, which is situate in the very centre of a poor and densely-populated district, was erected in 1888, the foundation-stone being laid by His Royal Highness the Duke of Cambridge, and was formally opened by His Grace the Duke of Westminster on April 23rd, 1890. It is chiefly intended for the treatment of accidents, which almost daily occur in the numerous factories and workshops with which this district abounds. The hospital provides two wards, one for men and another for women and children, besides a smaller ward for lads. In addition to these wards there is a separate ward reserved for the treatment of cases requiring isolation, the total number of beds being 38. How great the need was for a local hospital, is testified in the most conclusive manner by the fact that, within less than a month from the opening, every bed in the male ward was occupied.

"There can be no doubt that the hospital has already, during its short existence of three years, proved an inestimable boon to the labouring poor of this borough, the bulk of whom are engaged in more or less perilous occupations, while sufferers from all parts, far and near, have also been admitted. The institution is entirely free, accidents and cases of emergency are treated at once, and, if necessary, sent to the wards. All medical cases are attended in the out-patient department (the former dispensary, established in 1861) on the presentation of a letter of recommendation.

"During the last year 414 in-patients were admitted, while the out-patient department has been severely taxed, the average number of daily attendances being over 200. It has long been painfully evident that the present hospital accommodation is utterly inadequate to the growing requirements of the district, many cases having to be sent on to the London Hospital, which is three miles distant. The hope, however, of enlarging the hospital by adding another wing had, for lack of funds, to be abandoned, or at any rate to be deferred. The announcement, therefore, of Mr. Passmore Edwards having come forward with the munificent offer to bear the entire cost of building the much-needed wing for the accommodation of twenty more beds, was hailed by everybody in the borough with great joy and sincere gratitude.

"It must, however, not be overlooked that with the enlargement of the hospital, and the increase in the nursing and domestic staff, there will also be an increased annual expenditure of at least £1,500, which will bring the total expenditure of the institute to about £4,500. As the charity is unendowed, and entirely dependent on voluntary contributions for its support, it is obvious that the finances of the hospital ought to be put on a more stable basis than they are at present. It is the earnest desire of the committee that the good work of this charity may not be crippled for want of means, and they would most earnestly urge its claims, and recommend its welfare to the sympathy and generous support of their wealthy and benevolent friends in the West-end of London."

